# Access to lactation consult services during the COVID-19 pandemic and the impact on breastfeeding outcome variables

**DOI:** 10.1371/journal.pone.0318749

**Published:** 2025-03-18

**Authors:** Caroline Maltese, Chintan K. Gandhi, Sarah Ines Ramirez, Kristin K. Sznajder, Nicole Hackman

**Affiliations:** 1 Penn State College of Medicine, Hershey, Pennsylvania, United States of America; 2 Department of Pediatrics, Penn State College of Medicine, Hershey, Pennsylvania, United States of America; 3 Department of Family and Community Medicine, Penn State College of Medicine, Hershey, Pennsylvania, United States of America; 4 Department of Public Health Sciences, Penn State College of Medicine, Hershey, Pennsylvania, United States of America; King Saud University/Zagazig University, EGYPT

## Abstract

**Background**: Inpatient lactation consultation and social influences affect breastfeeding (BF) choices and sustainability. The COVID-19 pandemic introduced barriers to BF initiation and continuation including access to lactation support and social connection. Equitable access to lactation support can reduce health disparities.

**Research aim**: The study aimed to (1) determine the prevalence of professional lactation support during the COVID-19 pandemic, (2) explore the influence of this pandemic on the equitable accessibility to lactation support services, and (3) identify changes in BF rates and access to lactation support at three different phases of the pandemic (early, middle, and late).

**Methods**: Patients receiving prenatal care at a mid-sized academic medical institution in Central Pennsylvania were recruited and surveyed and this data was collected and combined with data from the electronic medical record.

**Results**: 88% of patients received a lactation consultation during birth hospitalization. Having COVID-19 during pregnancy did not change access to lactation consultation post-partum (p = 0.0961). Neither BF exclusivity during the three phases of the pandemic nor the number of lactation consult visits were statistically different (p = 0.2263; p = 0.0958 respectively). Multiple regression models assessing BF exclusivity in the hospital found significant associations with having a lactation consult (OR 2.50, 95% CI 1.04, 6.04), having an infant in the neonatal intensive care unit (OR 0.29, 95% CI 0.11, 0.73), and having reported social support during pregnancy (OR 1.09, 95% CI 1.01,1.18).

**Conclusions**: Social support during pregnancy and having a lactation consult visit during birth hospitalization remained critical factors for BF exclusivity. This study highlights the importance of having professional lactation support on both BF exclusivity and continuation during the COVID-19 pandemic.

## Introduction

Due to the numerous known health benefits of human milk, the American Academy of Pediatrics (AAP) and the World Health Organization (WHO) recommend exclusive breastfeeding until 6 months of age [1–3]. The anti-inflammatory, antimicrobial, and immunoregulatory effects of human milk strengthen the immune system of the child and decrease the risk of infection [1]. In addition, human milk feeding is associated with significantly lower risk of sudden infant death syndrome (SIDS), type 1 and 2 diabetes, and childhood obesity and it is linked to improved cognitive development [1]. Maternal health benefits are numerous, including accelerating uterine involution, reduction in the occurrence of postpartum hemorrhage and anemia, lactational amenorrhea, weight loss, and reduced risk of postpartum depression, type 2 diabetes, ovarian, breast, and endometrial cancer, osteoporosis, and hypertension [4].

In the setting of these recommendations, breastfeeding rates continue to be suboptimal. The National Immunization Survey conducted by the Center for Disease Control (CDC) showed that the rate of exclusive breastfeeding in the United States at 3 months was 45% [5]. By six months of age, rates of exclusive breastfeeding decreased to 25% [5]. These results highlight the need for additional and equitable support for patients who are breastfeeding for the lactating parent and baby to achieve ideal health [6]. Previous literature shows that both professional and personal support affects breastfeeding choices and sustainability [7,8]. International Board Certified Lactations Consultants (IBCLCs) are a source of professional support for breastfeeding individuals [3,9]. A systematic review from 2015 showed that IBCLC intervention significantly increased breast feeding initiation and breastfeeding duration, highlighting the crucial role of these professionals in successful adherence to the AAP and WHO guidelines [10].

Although the efficacy of these services has been studied, data on the prevalence of these services is limited. One study focused on improving breastfeeding rates for lactating parents with gestational diabetes prior to the pandemic found that 74.5% of their cohort received a lactation consult [11]. Other studies have shown that 77.8% of their participants had some contact during their hospitalization, although most of the contact was made after the initiation of feeding [12]. It has also been recorded that there are racial and ethnic disparities in both breastfeeding initiation and visitation by IBCLCs [12,13].

The coronavirus 2019 (COVID-19) pandemic resulted in barriers to breastfeeding and access to professional support [14]. One review article which explored the overall impact of COVID-19 on breastfeeding found that the breastfeeding hospital routine was altered with changes in guidelines concerning rooming in, dyad separation, and visitation practices [15]. Lactation care was affected as physical distancing requirements necessitated a shift from traditional, in-person education and care to virtual consultations. While some lactating parents opted for alternative feeding methods to human milk, other chose to breastfeed due to cost or difficulty accessing formula, belief that the formula could be contaminated, or belief that human milk provided the best immune benefits for their child [15]. The reduction in social networks due to isolation from family and friends further contributed to a lack of emotional and professional support for breastfeeding during this period [14,15].

The primary aim of the current study is to determine the prevalence of professional lactation support at a mid-sized academic medical institution in Central Pennsylvania during the COVID-19 pandemic. This study also aims to explore the influence of the pandemic on the equitable accessibility of lactation services. We will also investigate the rates of exclusive breastfeeding at the hospital and at one month postpartum in the setting of the COVID-19 pandemic. Lastly, this study aims to identify changes in breastfeeding and access to lactation support across three different phases of the pandemic (early, middle, and late).

## Materials and methods

### Research design

This prospective cohort study was part of the larger COVID-19 in Pregnancy Study, a longitudinal prospective study examining the impact of the COVID-19 pandemic on pregnant individuals and their newborns [16]. This type of analysis was used to compare woman with varying access to lactation support in the hospital and investigate the association to breastfeeding at the hospital and at 1 month postpartum. The study was approved by the institution’s Institutional Review Board under study number STUDY00014838.

### Setting and relevant context

In Pennsylvania (PA), located in the northeastern United States, 74.8% of children are ever breastfed, compared to the national rate of 83.2% and 24.6% are exclusively breastfeeding at 6 months which is just slightly below the national average (24.9%) [5]. According to the Maternity Practices in Infant Nutrition and Care (MPINC) Survey in 2022, PA scored above the national average for scores related to immediate postpartum care while the remainder of scores for rooming-in, feeding practices, feeding education & support and discharge support were consistent with the national averages [17]. In 2020, PA had a population of approximately 13 million, 80.8% of whom identified as white and 12% Black or African American [18]. The median household income in PA between 2018–2022 was $73,170 and almost 12% of people were estimated to live in poverty.

In this study, the pandemic was divided into three phases using a timeline of significant events provided by the CDC [19]. The first phase was defined from May 5, 2020, when data collection began, to November 3, 2020. At this time, pandemic stress was high, stay at home orders were in place, restrictions and limitations were established in the hospital systems, and patients experienced significant changes to family dynamics and stressors. The second phase was defined from November 4, 2020, to May 5, 2021. During this time vaccines became widely available to the public, restrictions were loosened, and small gatherings became more acceptable. The health system began to adapt to the pandemic by resuming elective procedures and optimizing socially distanced in-person care. The final phase of the pandemic was defined from May 6, 2021, to June 31, 2022. At this time, vaccination administration rates were increasing, social distancing became less pervasive, and patient care practices were returning to normal, despite the use of isolation practices.

### Sample

Patients who were at least 18 years of age, had access to the internet, were able to read English or Spanish, and were receiving prenatal care at an academic medical center in Central Pennsylvania were recruited for participation in this study. This institution is the main facility of a larger health system which spans 29 counties in Pennsylvania and includes four medical centers, three additional hospitals, 126 outpatient practices. The patient population comes from suburban and rural settings and is primarily white and middle class [16]. The system adheres to WHO’s ten steps to successful breastfeeding such as rooming-in, avoiding the use of commercial infant formulas in the early hours after births, and supports early initiation of breastfeeding [20]. Consistent with the CDC’s recommendation at that time, COVID-19 positive lactating parents were separated from their newborns to avoid the risk of transmission during the early phase of the pandemic [21]. Hospitalized post-partum COVID-19 positive lactating parents received lactation support both in person and via telephone, however, infants were not directly breastfed during the birth hospitalization.

Pregnant patients who gave birth to twins (*n* = 7) and those who did not have complete information on having a lactation support provider (*n* = 1), breastfeeding information at the birth hospital stay (n = 6) and breastfeeding information at one month postpartum (*n* = 7) for a total of 13 individuals with missing data from those three categories were excluded from the analysis in order to mitigate the confounders that could occur from a twin birth including birthweight and mode of delivery. In total, there were 230 people in the final dataset that combined data from the chart review, baseline survey during pregnancy, and the survey at one month postpartum. Participants received $25 at the end of their participation in the study at one month postpartum.

### Measurement

The survey data collected from this study was combined with information collected from a comprehensive chart review. Survey data included maternal information on age, marital status, education level, anxiety symptoms, depressive symptoms, social support, and breastfeeding at one month. Demographic characteristics were measured at baseline and mental health and social support questions were from both the baseline and one-month surveys and the breastfeeding question was from the one-month survey. Depressive symptoms were measured by the Patient Health Questionnaire-8 (PHQ8), anxiety symptoms were measured by the General Anxiety Disorder-7 (GAD7), and social support was measured using the Medical Outcomes Study modified 5-item social support survey (MSSS) [22–24]. Each item on the MSSS has five response options scored one through five “None of the time (1); A little of the time (2); Some of the time (3); Most of the time (4); All of the time (5)” [22]. The score for each of the five items is summed and a total score of up to 25 is calculated [22]. The lowest score of this scale is 5, indicating a low level of support [22]. The highest score and level of social support is given the numerical value of 25 [22]. Depressive symptoms were dichotomized as a score of 5 or greater on the PHQ8. Anxiety symptoms were considered high based on a score of ten or greater on the GAD7.

### Data collection

Patients were recruited through email, mail, phone calls, social media, and fliers and the electronic medical record. Women were consented by telephone or an email consent document. Verbal consent was obtained by a study team member using the telephone and eligibility script approved by the IRB. Verbal consent was obtained from women who elected to answer survey questions over the phone. Implied consent was obtained from women who are recruited via mail, email, or phone and choose to complete the surveys online themselves. If consent was obtained by telephone, consenters used a program called MAX to hide their personal telephone number.

Enrollment began in May 2020 and was completed in June 2022. The survey data collected during pregnancy and at one month postpartum were included in this analysis. A comprehensive chart review was conducted from June 2022 until August 2022, and we extracted information on race, ethnicity, gravidity, parity, COVID-19 diagnosis during pregnancy, birth weight, gestational age, mode of delivery, an admission to the neonatal intensive care unit (NICU), previous breastfeeding experience, breastfeeding at the hospital, lactation consult while in the hospital, number of lactation consultations, and time to the lactation consult in the hospital. Authors did not have access to information that could identify individual participants after data collection was completed.

### Data analysis

Due to the majority of participants in the dataset being white, the race category was dichotomized as white compared with non-white. Parity was dichotomized as primiparous compared with multiparous. Birth weight was analyzed as a continuous variable. Gestational age was dichotomized at less than 37 weeks (preterm and late preterm) compared with at least 37 weeks (early term and full term). Mode of delivery was dichotomized as vaginal delivery compared with cesarean delivery. The number of lactation consultations were categorized as none, one, and two or more. The time to lactation consultation was kept as a continuous variable.

Descriptive statistics including frequency counts for categorical variables and mean and standard deviations for continuous variables were reported. Bivariate associations were computed using Chi Square or ANOVA tests for categorical variables, Fisher’s Exact tests for categorical variables with a cell count of five or less, and t-tests for continuous exposure variables. Multivariable logistic regression models examining the social determinants of health, including perceived social support, prenatal and postnatal anxiety and depressive symptoms and breastfeeding at 1 month postpartum were completed by including variables with statistically significant (i.e., p<0.05) bivariate associations.

## Results

In total, 230 participants who delivered at an academic medical center in Central Pennsylvania were included in the study. Demographic information for pregnant patients who had a lactation consult compared to those pregnant patients who did not have a lactation consult during birth hospitalization can be found in Table 1. There was no statistically significant difference in race, age, birthweight, education, marital status, length of term, neonatal intensive care unit (NICU) stay, COVID-19 during pregnancy, breastfeeding at the hospital or at one month, prenatal and postpartum anxiety symptoms, depressive symptoms, and social support and previous breastfeeding experience between those who received a lactation consult and those who did not have a lactation consult. Primiparous pregnant patients and those who delivered via C-section were more likely to have a lactation consult. Utilization of lactation services during birth hospitalization did not vary based on high versus low level of prenatal depressive or anxiety symptoms or social support during pregnancy.

**Table 1:** Population characteristics and their association with receiving a lactation consult.

	Total *n* = 230 *n* (%) Mean (SD)	Pregnant patients who had a lactation consult (*n* = 203, 88.3%) *n* (row %) Mean (SD)	Pregnant patients who did not have a lactation consult (*n* = 27, 11.7%) *n* (row %) Mean (SD)	P-value
Age	31.5 (4.8)	31.5 (+4.9)	31.9 (+4.4)	0.6663
Race White Non-white	205 (89.1) 25 (10.9)	181 (88.3) 22 (88.0)	24 (11.7) 3 (12.0)	1.0000
Education At least some college Completed a college degree or more 58 (25.3) 171 (74.7)	50 (86.2) 152 (88.9)	8 (13.8) 19 (11.1)	0.5842	
Marital status Married Not Married	190 (82.6) 40 (17.4)	167 (87.9) 36 (90.0)	23 (12.1) 4 (10.0)	1.0000
Parity Primiparous Multiparous	99 (43.0) 131 (56.9)	95 (96.0) 108 (82.4)	4 (4.0) 23 (17.6)	0.0016
COVID-19 during pregnancy Yes No	10 (4.4) 220 (96.0)	10 (100.0) 193 (87.7)	0 27 (12.3)	0.6113
Mode of delivery Vaginal birth C-section	172 (74.8) 58 (25.2)	146 (84.9) 57 (98.3)	26 (15.1) 1 (1.7)	0.004024
Length of gestation Preterm Full term	13 (5.7) 217 (94.3)	12 (92.3) 191 (88.0)	1 (7.7) 26 (12.0)	1.0000
Baby weight (g)	3348.1 (+579.9)	3357.0 (+600.9)	3281.0 (+389.5)	0.5237
Admission to NICU Yes No	23 (10.1) 204 (89.9)	22 (95.7) 178 (87.3)	1 (4.3) 26 (12.7)	0.3259
Exclusive BF in hospital Yes No	176 (76.5) 54 (23.5)	159 (90.3) 44 (81.5)	17 (9.7) 10 (18.5)	0.1997
Exclusive BF at one month postpartum Yes No	145 (63.0) 85 (37.0)	131 (90.3) 72 (84.7)	14 (9.7) 13 (15.3)	0.2095
Prenatal anxiety symptoms (GAD7) High (>10) Low	7 (7.6) 85 (92.4)	6 (85.7) 73 (85.9)	1 (14.3) 12 (14.1)	1.0000
Prenatal depressive symptoms (PHQ8) High(>5) Low	59 (26.1) 167 (73.9)	49 (83.1) 150 (89.8)	10 (16.9) 17 (0.2)	0.1682
Social support in pregnancy*	20.9 (+3.9)	20.9 (+4.0)	20.9 (+3.7)	0.8655
Previous breastfeeding experience Yes No	104 (51.5) 98 (48.5)	104 (100.0) 94 (95.9)	0 4 (4.1)	0.0536
Time in the pandemic May 2020–Oct 2020 Nov 2020–April 2021 May 2021–June 2022 136 (59.1)	40 (17.4) 54 (23.5)	122 (89.7) 34 (85.0) 47 (87.0)	14 (10.3) 6 (15.0) 7 (12.9)	0.6830

* Medical Outcomes Study modified 5-item social support survey (MSSS). A score of 5 indicates the lowest level social support and a score of 25 indicates the highest level of social support.

An increased prevalence of exclusive breastfeeding during the birth hospital stay was associated with patients who had completed a college degree or more, were married, and whose newborn had a higher birth weight, no NICU stay, and reported higher levels of social support in pregnancy (Table 2). There were no statistically significant differences in race, age, route of delivery, parity, COVID-19 during pregnancy, length of gestation, having previous breastfeeding experience, prenatal anxiety and depressive symptoms, postpartum depressive symptoms, number of consults or time to lactation consult between the two study groups. There was no statistically significant difference in exclusive breastfeeding in the hospital throughout the three phases of the pandemic, however exclusivity tended to be higher during the early lockdown phase when compared to late pandemic (80.1% vs 68.5%).

**Table 2:** Population characteristics and their association with exclusive breastfeeding in the hospital.

	Total *n* = 230 *n* (%)	Exclusive BF at hospital (*n* = 176, 76.5%) *n* (row %)	No exclusive BF at hospital (*n* = 54, 23.5%) *n* (row %)	P-value
Age	31.5 (4.8)	31.2 (+4.8)	32.3 (+4.9)	0.1628
Race White Non-white	205 (89.1) 25 (10.9)	159 (77.6) 17 (68.0)	46 (22.4) 8 (32.0)	0.2870
Education At least some college completed Completed a college degree or more	58 (25.3) 171 (74.7)	37 (63.8) 139 (81.3)	21 (36.2) 32 (18.7)	0.0063
Marital status Married Not Married	190 (82.6) 40 (17.4)	151 (79.5) 25 (62.5)	39 (20.5) 15 (37.5)	0.0213
Parity Primiparous Multiparous	99 (43.0) 131 (56.9)	80 (80.8) 96 (73.3)	19 (19.2) 35 (26.7)	0.1825
COVID-19 during pregnancy Yes No	10 (4.4) 220 (96.0)	8 (80.0) 168 (76.4)	2 (20.0) 52 (23.6)	1.0000
Mode of delivery Vaginal birth C-section	172 (74.8) 58 (25.2)	136 (79.1) 40 (68.9)	36 (20.9) 18 (31.0)	0.1164
Length of gestation Preterm Fullterm	13 (5.7) 217 (94.3)	8 (61.5) 168 (77.4)	5 (38.5) 49 (22.6)	0.1909
Birth weight (g)	3348.1 (+579.9)	3400.5 (+519.9)	3177.2 (+722.4)	0.0130
Admission to NICU Yes No	23 (10.1) 204 (89.9)	12 (52.2) 161 (78.9)	11 (47.8) 43 (21.1)	0.0043
Prenatal anxiety symptoms (GAD7) High (>10) Low	7 (7.6) 85 (92.4)	5 (71.4) 59 (69.4)	2 (28.6) 26 (30.6)	1.0000
Prenatal depressive symptoms (PHQ8) High (>5) Low	59 (26.1) 167 (73.9)	44 (74.6) 128 (76.7)	15 (25.4) 39 (23.3)	0.7485
Social support in pregnancy*	20.9 (+3.9)	21.4 (+3.7)	19.5 (+4.4)	0.0020
Time in the pandemic May 2020–Oct 2020 Nov 2020–April 2021 May 2021–June 2022	136 (59.1) 40 (17.4) 54 (23.5)	109 (80.1) 30 (75.0) 37 (68.5)	27 (19.1) 10 (33.3) 17 (31.5)	0.2263
Number of consults 1 2 More than 2	89 (43.8) 65 (32.0) 49 (21.1)	72 (80.9) 54 (83.1) 33 (67.4)	17 (19.1) 11 (16.9) 16 (32.7)	0.0958
Time to consult (in hours from delivery)	17.5 (+10.3)	17.3 (+10.4)	18.1 (+10.0)	0.6517
Previous breastfeeding experience Yes No	104 (51.5) 98 (48.5)	83 (79.8) 75 (76.5)	21 (20.2) 23 (23.5)	0.5728
Lactation consultation No lactation consultation	203 (88.3) 27 (11.8)	159 (78.3) 17 (62.9)	44 (21.7) 10 (37.0)	0.0768

* Medical Outcomes Study modified 5-item social support survey (MSSS). A score of 5 indicates the lowest level social support and a score of 25 indicates the highest level of social support.

When a multiple regression model was used to analyze the statistically significant variables in the bivariate analysis, lactation consult during birth hospital stay and NICU stay had the largest effect on exclusive breastfeeding at birth hospitalization (Table 3).

**Table 3:** Logistic regression of variables significant in the bivariate results and the association with exclusively breastfeeding at the birth hospital stay.

Variable	Bivariate	Multiple	Forward selection
Completed a college degree or more	2.47 (1.28, 4.77)	1.85 (0.85, 4.02)	–
Married	2.32 (1.12, 4.82)	1.29 (0.53, 3.13)	–
Birthweight (g)	1.00 (1.00, 1.00)	1.00 (1.00, 1.00)	–
NICU	0.29 (0.12, 0.71)	0.49 (0.18, 1.39)	0.29 (0.11, 0.73)
Social support in pregnancy	1.12 (1.04, 1.20)	1.09 (1.00, 1.18)	1.09 (1.01, 1.18)
Lactation consult	2.13 (0.91, 4.97)	2.32 (0.94, 5.73)	2.50 (1.04, 6.04)

The demographic information for pregnant patients who exclusively breastfed at one month compared to those pregnant patients who did not exclusively breastfeed at one month can be found in Table 4. There were no statistically significant differences in race, age, birthweight, marital status, route of delivery, parity, length of term, COVID-19 during pregnancy, prenatal anxiety and depressive symptoms, social support in pregnancy and postpartum, having had previous breastfeeding experience, or receiving a lactation consultation between the two groups. Again, there was no difference in exclusive breastfeeding at one month throughout the three phases of the pandemic. A higher prevalence of exclusive breastfeeding at one month was associated with having a college degree or more, no NICU stay, low postpartum anxiety and depressive symptoms, and having had exclusive breastfeeding in the birth hospital stay.

**Table 4:** Population characteristics and their association with breastfeeding at one month postpartum.

	Total n = 230 n (%)	Exclusive BF at one month (n = 145, 63.0%) n (row %)	Not exclusively BF at one month (n = 85, 37.0%) n (row %)	P-value
Age	31.5 (4.8)	31.2 (+4.6)	31.9 (+5.2)	0.3137
Race White Not white	205 (89.1) 25 (10.9)	131 (63.9) 14 (56.0)	74 (36.1) 11 (44.0)	0.4396
Education At least some college completed Completed a college degree or more	58 (25.3) 171 (74.7)	26 (44.8) 119 (69.6)	32 (55.2) 52 (30.4)	0.0007
Marital status Married Not Married	190 (82.6) 40 (17.4)	123 (64.7) 22 (55.0)	67 (35.3) 18 (45.0)	0.2462
Parity primiparous Multiparous	99 (43.0) 131 (56.9)	61 (61.6) 84 (64.1)	38 (38.4) 47 (35.9)	0.6966
COVID-19 during pregnancy Yes No	10 (4.4) 220 (96.0)	9 (9.0) 136 (61.8)	1 (10.0) 84 (38.2)	0.0961
Mode of delivery Vaginal birth C-section	172 (74.8) 58 (25.2)	113 (65.7) 32 (55.2)	59 (34.3) 26 (44.8)	0.1510
Length of gestation Preterm Full term	13 (5.7) 217 (94.3)	9 (69.2) 136 (55.2)	4 (30.8) 81 (37.3)	0.7721
Baby weight	3348.1 (+579.9)	3357.2 (+596.6)	3332.5 (+553.4)	0.7558
Admission to NICU Yes No	23 (10.1) 204 (89.9)	10 (43.5) 133 (65.2)	13 (56.5) 71 (34.8)	0.0409
Prenatal anxiety symptoms (GAD7) High (>10) Low	7 (7.6) 85 (92.4z)	4 (57.1) 52 (61.2)	3 (42.9) 33 (38.8)	1.0000
Prenatal depressive symptoms (PHQ8) High(>5) Low	59 (26.1) 167 (73.9)	33 (55.9) 109 (65.3)	26 (44.1) 58 (34.7)	0.2020
Postpartum anxiety symptoms (GAD7) High(>10) Low	10 (5.9) 159 (94.1)	3 (30.0) 105 (66.0)	7 (70.0) 54 (34.0)	0.0368
Postpartum depressive symptoms (PHQ8) High (>5) Low	38 (17.1) 184 (82.9)	17 (44.7) 124 (67.4)	21 (55.3) 60 (32.6)	0.0083
Social support in pregnancy*	20.9 (+3.9)	21.2 (+3.8)	20.6 (+4.2)	0.3019
Social support postpartum*	21.1 (+3.8)	21.2 (+3.7)	20.7 (+3.9)	0.2750
Exclusive breastfeeding in the hospital Yes No	176 (76.5%) 54 (23.5%)	132 (75.0) 13 (24.1)	44 (25.0) 41 (75.9)	<0.0001
Time in the pandemic May 2020–Oct 2020 Nov 2020–April 2021 May 2021–June 2022	136 (59.1) 40 (17.4) 54 (23.5)	85 (62.5) 30 (75.0) 30 (55.6)	51 (37.5) 10 (25.0) 24 (44.4)	0.1517
Lactation consult No lactation consult	203 (88.3) 27 (11.8)	131 (64.5) 14 (51.9)	72 (35.5) 14 (48.2)	0.1997
	Total n = 230 n (%)	Exclusive BF at one month (n = 145, 63.0%) n (row %)	Not exclusively BF at one month (n = 85, 37.0%) n (row %)	P-value
Number of consults 1 2 More than 2	89 (43.8) 65 (32.0) 49 (21.1)	64 (71.9) 37 (56.9) 30 (55.6)	25 (28.1) 28 (43.1) 19 (38.8)	0.1357
Time to consult	17.5 (+10.3)	17.1 (+9.5)	18.2 (+11.6)	0.4944
Previous breastfeeding experience Yes No	104 (51.5) 98 (48.5)	71 (68.3) 58 (59.2)	33 (31.7) 40 (40.8)	0.1791

* Medical Outcomes Study modified 5-item social support survey (MSSS). A score of 5 indicates the lowest level social support and a score of 25 indicates the highest level of social support.

When a multiple regression model was used to analyze the statistically significant variables, exclusive breastfeeding at the hospital had the largest impact on exclusive breastfeeding at one month (Table 5).

**Table 5:** Logistic regression of variables significant in the bivariate results at 1 month and association with exclusively breastfeeding at 1 month.

Variable	Bivariate	Multiple	Forward selection
Completed a college degree or more	2.82 (1.53, 5.19)	2.56 (1.07, 6.09)	2.69 (1.21, 5.97)
NICU	0.41 (0.17, 0.98)	1.18 (0.29, 4.70	–
High postpartum anxiety symptoms	0.22 (0.06, 0.89)	0.99 (0.16, 6.13)	–
High postpartum depressive symptoms	0.39 (0.19, 0.79)	0.55 (0.21, 1.45)	–
Exclusive breastfeeding in the hospital	9.46 (4.65, 19.27)	7.91 (3.46, 18.07)	8.11 (3.63, 18.13)
Lactation consult	1.69 (0.75, 3.79)	1.32 (0.43, 4.01)	–

## Discussion

The primary aim of this analysis was to determine the prevalence of professional lactation support at a mid-sized academic medical institution in Central Pennsylvania during the COVID-19 pandemic. Our results reveal that 88.3% of the patients included in the study received a lactation consultation, a greater percentage than what has previously been reported in the literature, despite the challenges associated with the COVID-19 pandemic [11–13]. Primiparous patients and patients who underwent a C-section delivery were more likely to have a lactation consult during this period compared to multiparous patients and those who had a vaginal delivery.

As this study aimed to explore the influence of the pandemic on the equitable accessibility of lactation services, there were no statistically significant differences in race, education, marital status, age, or history of COVID-19 in pregnancy for patients who received a lactation consult compared to those who did not, highlighting the equitable delivery of this valuable service, despite the pandemic. Consistent with prior literature in the field, we add additional evidence demonstrating the power of lactation support during the pandemic to increase breastfeeding exclusivity in the hospital and at one month postpartum [25] We found significant associations of a lactation consult, having an infant in the NICU, and social support during pregnancy with breastfeeding exclusivity during the birth hospital stay. Breastfeeding continuation at one month postpartum was significantly associated with exclusive breastfeeding in the hospital, and having at least a college degree, which is consistent with literature published during non-pandemic periods [26–28].

Historically, the role of NICU admission on breastfeeding outcome variables has been found to be inconsistent; with some publications reporting NICU admission having a positive association with breastfeeding initiation for certain preterm infants while others reported decreased likelihood of breastfeeding continuation [29,30]. This current analysis adds to the literature surrounding the COVID-19 pandemic, finding the exposure to the NICU setting during the pandemic negatively impacted breastfeeding initiation in the hospital in this cohort. We theorize that during the pandemic, infants in the NICU setting had limited parental presence and reduced social support due to implemented restrictions to minimize the likelihood of NICU infants and staff contracting COVID-19.

Our results are consistent with current evidence that breastfeeding support interventions improved exclusive breastfeeding rates [10,25]. Additionally, it has been previously documented that C-section delivery compared to vaginal delivery has adverse effects on breastfeeding variables, which would necessitate an increased amount of lactational support [31,32]. Support for breastfeeding, including professional lactation support, and social support from family and friends, has been shown to affect breastfeeding choices and sustainability, which remained consistent for patients who gave birth during the pandemic [7,8]. Our data that advanced education is associated with higher rates of breastfeeding at one month postpartum is consistent with the previously documented relationship between higher odds of early initiation of breastfeeding among the lactating parents with advanced education [26–28]. Lastly, high postpartum anxiety and depressive symptoms were associated with reduced rates of exclusive breastfeeding at one month in this cohort, consistent with previous data showing that postpartum patients who experience symptoms of anxiety are at an increased risk of suboptimal infant-feeding outcomes. This relationship highlights the importance of improving identification and treatment of both maternal postpartum anxiety and depressive symptoms to optimize both maternal and infant health outcomes [33].

The final aim of this study was to identify changes in breastfeeding and access to lactation support across three different phases of the pandemic. The pandemic created additional barriers to meeting breastfeeding goals, as access to lactation support providers and participation in prenatal and breastfeeding classes were significantly reduced, or transitioned to virtual experiences and families were experiencing increased levels of stress [14]. A Morbidity and Mortality Weekly Report produced by the CDC showed that 17.9% of hospitals offered decreased in person lactation support and often patients were discharged much faster than in non-pandemic times [34]. However, this study indicates that equitable professional lactation support was still being provided to pregnant patients during the pandemic and remained effective in increasing rates of exclusive breastfeeding both at the birth hospitalization and at one month postpartum. This is consistent with the CDC report which showed that 68.9% of the hospitals included in the analysis reported that exclusive breastfeeding rates during birth hospitalization stayed the same [34].

This study adds to existing knowledge by documenting breastfeeding trends during the COVID-19 pandemic. While early pandemic breastfeeding exclusivity was higher than later in the pandemic, our results did not indicate a statistically significant difference in outcomes between the three phases of the pandemic, showing that the initial setbacks and challenges encountered by health systems did not have significant detrimental effects on breastfeeding outcomes and were perhaps mitigated by the benefits of stay-at-home policies. Longer term monitoring of breastfeeding rates and childhood development will be important to further understand the impact of the pandemic on overall infant health.

## Limitations

This analysis was limited by the small sample size that was composed of participants recruited from one institution. Consistent with participation studies where participants tended to have higher levels of education and income, our study participants were generally white, non-Hispanic, well-educated women, which limits generalization of our findings [27]. Additionally, the study had only included breastfeeding outcomes at two points in time (at the hospital and 1 month), which may not reflect long term exclusive breastfeeding outcomes. For example, there is evidence that lactating parents experienced increased stress and higher depressive symptoms scores in the 3rd month of breastfeeding, a data point that was not analyzed in this study [35]. Professional lactation efforts during the pandemic varied widely across the globe, so the effects on breastfeeding outcomes could have significant variability.

## Conclusion

Breastfeeding has enormous health benefits for infants and their lactating parents and equitable access to lactation support is critical to reducing health disparities. This study highlights that the COVID-19 pandemic did not negatively impact the equitable access to lactation services or exclusive breastfeeding at the hospital or at one month. This study highlights the importance of receiving lactation consultation to increase breastfeeding exclusivity and continuation both in pandemic and non-pandemic times. Increasing access to lactation support providers and social support during pandemic periods has the potential to reduce health disparities in our communities.
